# Negative threshold voltage shift in an a-IGZO thin film transistor under X-ray irradiation†

**DOI:** 10.1039/c9ra03053k

**Published:** 2019-07-03

**Authors:** Dong-Gyu Kim, Jong-Un Kim, Jun-Sun Lee, Kwon-Shik Park, Youn-Gyoung Chang, Myeong-Ho Kim, Duck-Kyun Choi

**Affiliations:** Division of Materials Science and Engineering, Hanyang University 222, Wangsimni-ro, Seongdong-gu Seoul Republic of Korea duck@hanyang.ac.kr; Research and Development Center, LG Display Co., Ltd. 30, Magokjungang 10-ro, Gangseo-gu Seoul Republic of Korea

## Abstract

We investigated the effects of X-ray irradiation on the electrical characteristics of an amorphous In–Ga–Zn–O (a-IGZO) thin film transistor (TFT). The a-IGZO TFT showed a negative threshold voltage (*V*_TH_) shift of −6.2 V after 100 Gy X-ray irradiation. Based on spectroscopic ellipsometry (SE) and X-ray photoelectron spectroscopy (XPS) analysis, we found that the Fermi energy (*E*_F_) changes from 2.73 eV to 3.01 eV and that the sub-gap state of D1 and D2 changes near the conduction band minimum (CBM) of the a-IGZO film after X-ray irradiation. These results imply that the negative *V*_TH_ shift after X-ray irradiation is related to the increase in electron concentration of the a-IGZO TFT active layer. We confirmed that the sources for electron generation during X-ray irradiation are hydrogen incorporation from the adjacent layer or from ambient air to the active layer in the TFT, and the oxygen vacancy dependent persistent photocurrent (PPC) effect. Since both causes are reversible processes involving an activation energy, we demonstrate the *V*_TH_ shift recovery by thermal annealing.

## Abstract 2

We studied the effect of X-ray irradiation on the negative threshold voltage shift of bottom-gate a-IGZO TFT. Based on spectroscopic analyses, we found that this behavior was caused by hydrogen incorporation and oxygen vacancy ionization.

## Graphical abstract

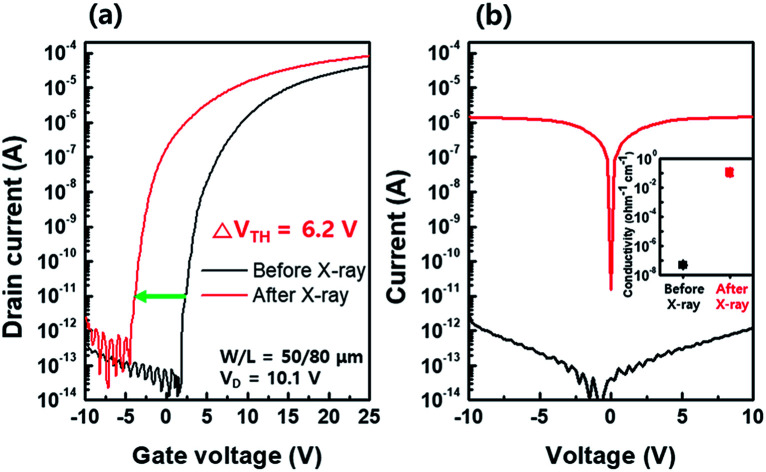

## Introduction

Recently, digital X-ray detectors (DXDs) have attracted attention due to their information storage ability and rapid information transmission compared to film-based analog X-ray detectors. DXDs are classified as direct- and indirect-types, depending on the method of converting the X-ray into an electric signal. Indirect-type DXDs have advantages regarding power consumption, noise power spectrum (NPS), and detective quantum efficiency (DQE) compared with direct-type DXDs.^1–3^ In indirect-type DXD panels, each pixel has three basic elements: a thin film transistor (TFT), a photodiode, and a scintillation layer that converts X-rays to visible light on the top.^3,4^

Many researchers have intensively studied dynamic image DXDs applicable for medical and industrial usage. For these purposes, TFTs in the DXD panels must meet specifications such as low leakage current and high mobility to reduce the signal-to-noise ratio (SNR) and prevent image delay.^4,5^ The typical active layer material of the TFTs used in DXD panels has been a-Si : H, which has limited ability to drive dynamic images due to its high leakage current and low mobility (<0.5 cm^2^ V^−1^ s^−1^).^3–6^ Meanwhile, oxide-based TFTs are known to have excellent electrical characteristics compared with a-Si : H TFTs, such as low leakage current, high mobility (>10 cm^2^ V^−1^ s^−1^), and a high on-off ratio.^6–8^ Therefore, oxide-based TFTs in DXD panels are a promising solution for dynamic imaging systems. However, since many studies have reported that the oxide-based TFTs are not reliable under light illumination, it is necessary to study the effect of higher energy X-rays on the stability.^9–11^ In particular, such an evaluation is essential for the industrial DXD panels where the X-ray irradiation condition is severe.

The most concerning electrical property in oxide-based TFTs is the *V*_TH_ shift that can cause a malfunction of device. The commonly observed negative *V*_TH_ shift under various stress conditions is known to be associated with the increase in electron concentration in the active layer. One of the plausible reasons for this effect is hydrogen incorporation. Hydrogen can exist as shallow donor-like states in the form of interstitial hydrogen (H_i_^+^) or substitutional hydrogen (H_O_^+^) in oxide semiconductors.^12,13^ However, based on a first principle calculation, a few groups claimed that deep H-related centers like interstitial hydrogen (H_i_^−^) and substitutional hydrogen (H-DX^−^) are also energetically available.^14,15^ In particular, Kang *et al.* also reported that visible light can excite the electron transition of H_i_^−^ and H-DX^−^ states to H_i_^+^ and H_O_^+^ states, leading to increases in the electron concentration of oxide semiconductors.^15^ In addition to the hydrogen associated electron generation, another path to generate electrons is designated as the persistent photocurrent (PPC) effect. Visible light can cause the transition of the neutral oxygen vacancy (V_O_) state to the charged oxygen vacancy (V_O_^2+^) state, and simultaneously forms delocalized electrons in the conduction band.^9–11^

The *V*_TH_ shift and other electrical characteristics in the oxide-based TFTs under the various biased or illumination conditions have been studied by a number of researchers as mentioned previously. However, the X-ray irradiation on the electrical characteristics, especially on *V*_TH_, has not been explored to date. The shift in *V*_TH_ would be the most serious concern in industrial applications where a high dose or a long-term X-ray illumination environment is inevitable. In this study, we examined the effect of X-ray irradiation on the electrical characteristics of a bottom-gated a-IGZO TFT. To investigate the effect of X-rays more precisely, we prepared thin film type a-IGZO samples in addition to a-IGZO transistors. The behavior and effect of hydrogen incorporation and oxygen vacancies under X-ray irradiation were evaluated by spectroscopic ellipsometry (SE) and X-ray photoelectron spectroscopy (XPS) analyses.

## Experimental

**Fig. 1:**
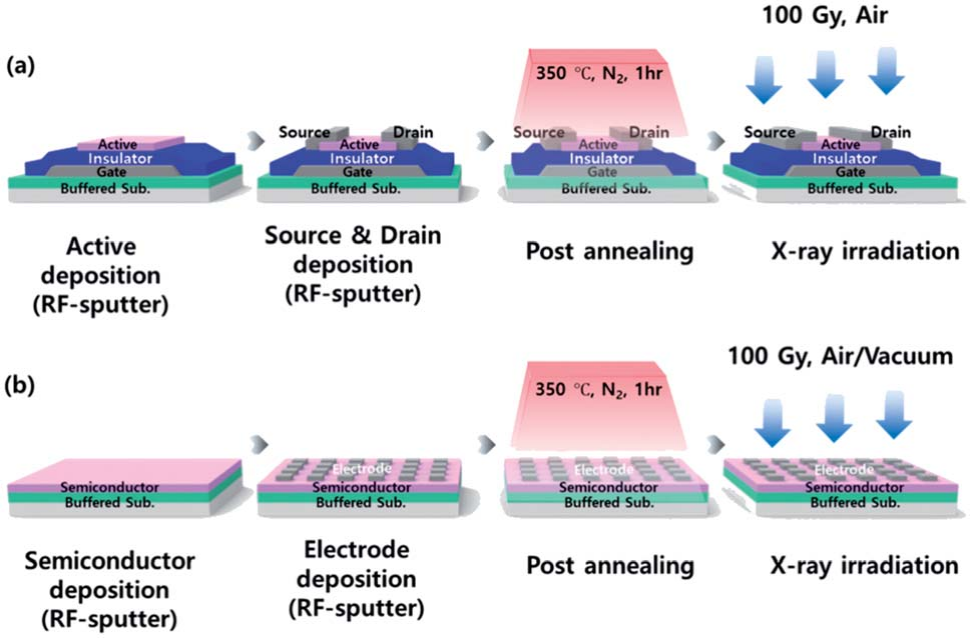
Schematic flows of the fabrication of (a) a bottom-gated amorphous In–Ga–Zn–O (a-IGZO) thin film transistor (TFT) and (b) film type a-IGZO sample. In the device, Mo is used as a gate, SiO_2_ is used as a gate insulator, a-IGZO is used as an active layer (semiconductor), and In–Sn–O (ITO) is used as source and drain top electrodes.

The Mo bottom electrode was deposited by DC sputtering on a SiN_*X*_/SiO_2_ buffered glass substrate and was patterned using photolithography process. A 100 nm-thick SiO_2_ gate insulator was deposited using plasma-enhanced chemical vapor deposition (PECVD) at 350 °C. As an active layer, 40 nm-thick a-IGZO (In : Ga : Zn = 1 : 1 : 1 mol%) film was deposited by RF sputtering at 40 W, at a working pressure of 4.5 mTorr, and the Ar : O_2_ ratio of 9 : 1. After the active layer was defined, a 100 nm-thick In–Sn–O (ITO) (10 wt% SnO_2_) transparent top electrode was deposited by RF sputtering. The source and drain area were defined and patterned by a lift-off process. Finally, a post-annealing process was performed for 1 h at 350 °C under an ambient N_2_ atmosphere. The width and length ratio (*W*/*L*) of a-IGZO TFT was 50/80 μm. In addition to the TFT device fabrication, film type a-IGZO samples were also prepared to evaluate the current level change of the a-IGZO active layer more clearly using identical process condition. The electrode size of a-IGZO film was 100 × 100 μm. The fabrication process of the a-IGZO TFT and a-IGZO film are presented in Fig. 1(a) and (b). In addition, the samples for spectroscopic ellipsometry (SE) analysis was fabricated by depositing a 40 nm thick a-IGZO film on silicon substrate in which used to acquire an existing database in the analysis equipment. Whereas samples for X-ray photoelectron spectroscopy (XPS) used a glass substrate with a buffer layer.

For X-ray irradiation, a TVX-IL3205 device from Tech Valley corporation was used at a dose rate of 0.66 Gy min^−1^. The typical exposure time was adjusted to 2 h and 3 min to set the total X-ray dose to 100 Gy. An Agilent E5270B parametric analyzer was used to evaluate the electrical characteristics of the a-IGZO TFTs and a-IGZO thin films. Spectroscopic ellipsometry (SE, RC2®) and X-ray photoelectron spectroscopy (XPS, Phi 5000 VErsaProbe) were used to analyze the defect states near the conduction band of a-IGZO and to investigate the oxygen chemical states, respectively.

## Results and discussion

**Fig. 2:**
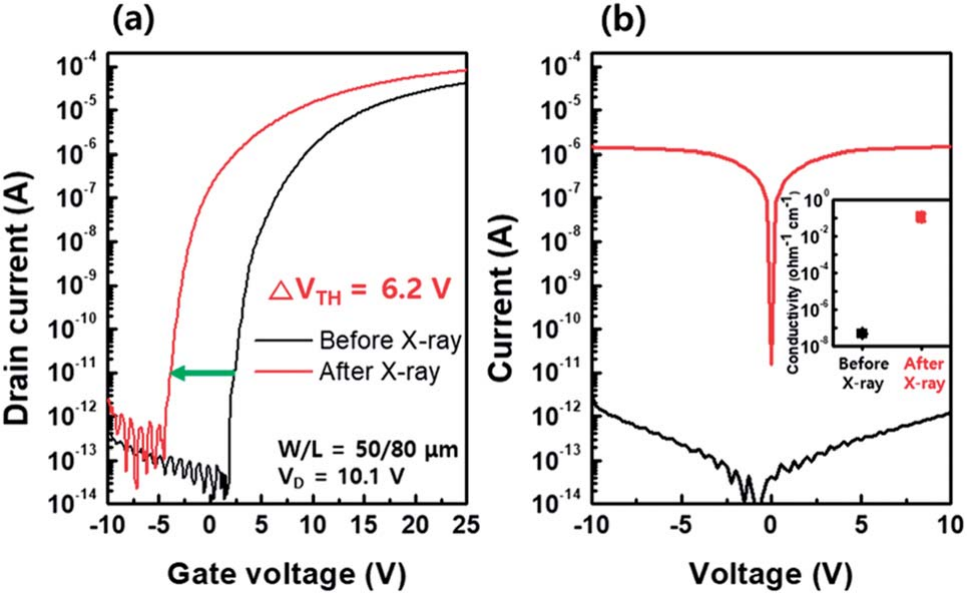
(a) Transfer characteristics of an amorphous In–Ga–Zn–O (a-IGZO) thin film transistor (TFT) before (black line) and after (red line) X-ray irradiation. (b) Increase in current level and conductivity (inset) of the active layer before (black line) and after (red line) X-ray irradiation.

Fig. 2(a) shows the transfer characteristics of the bottom-gated a-IGZO TFT before (black line) and after (red line) X-ray irradiation. The initial a-IGZO TFT showed a field-effect mobility (*μ*_fe_) of 15.3 cm^2^ V^−1^ s^−1^, threshold voltage (*V*_TH_) of 1.8 V, and sub-threshold swing (S/S) of 0.42 V per decade. However, the a-IGZO TFT exposed to 100 Gy X-ray doses resulted in a *μ*_fe_ of 17.1 cm^2^ V^−1^ s^−1^, *V*_TH_ of −4.4 V, and S/S of 0.42 V per decade. It showed significant negative *V*_TH_ shift of −6.2 V after X-ray irradiation. We independently measured the current level of the active layer by probing the source and drain electrodes on the same device, as shown in Fig. 2(b); a 6 orders of magnitude increase in the current level of the a-IGZO active layer was observed after X-ray irradiation. This result implies that the negative *V*_TH_ shift of the a-IGZO TFT mainly relies on the conductivity change in the active layer during X-ray irradiation.

**Fig. 3:**
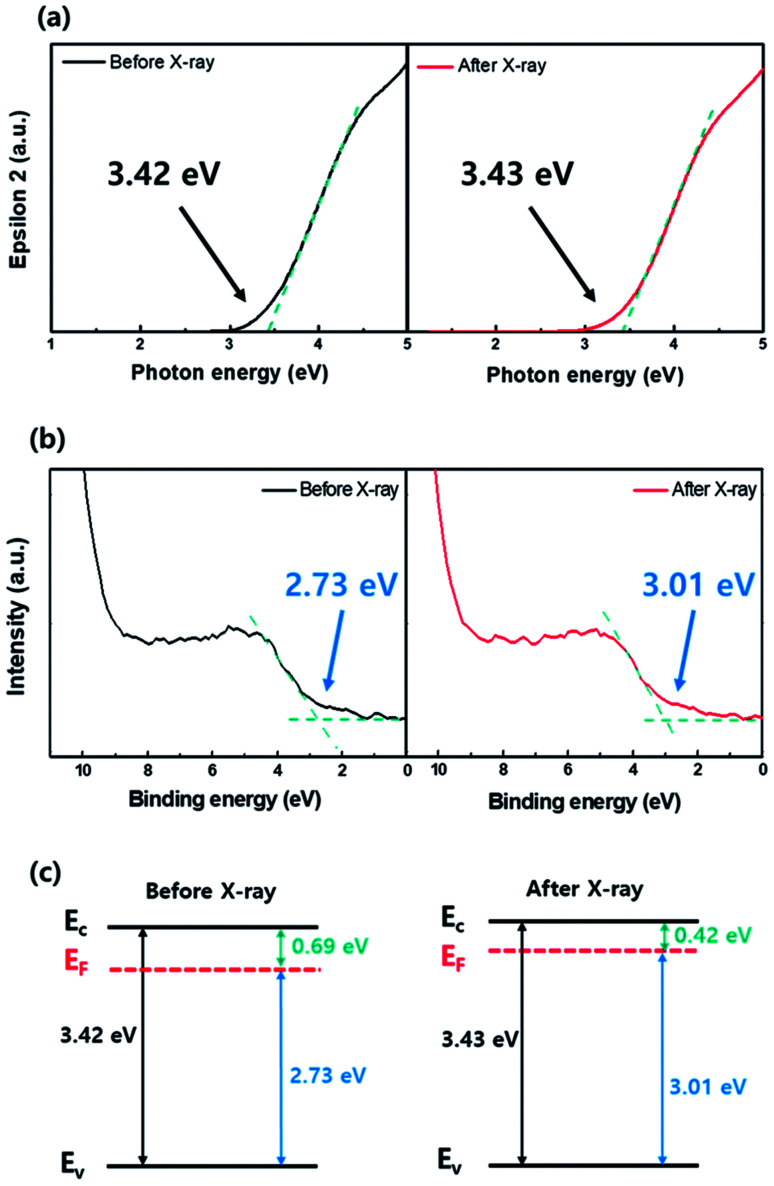
(a) Dielectric function (*ε*_2_) change of an amorphous In–Ga–Zn–O (a-IGZO) film before and after X-ray irradiation analyzed from the spectroscopic ellipsometry (SE) spectra. The band gap energy of the a-IGZO film is determined using this analysis. (b) Fermi energy level change of the a-IGZO film determined from the X-ray photoelectron spectroscopy (XPS) valence band edge analysis. (c) Corresponding band alignment of the a-IGZO film before and after X-ray irradiation by integrating the band gap energy and Fermi energy level in (a) and (b).

To understand the conductivity changes in the active layer during X-ray irradiation, spectroscopic ellipsometry (SE) and X-ray photoelectron spectroscopy (XPS) analyses were performed on the separately prepared film type a-IGZO specimen. Fig. 3(a) and (b) present the bandgap (*E*_g_) and the Fermi energy (*E*_F_) level of a-IGZO film before and after X-ray irradiation, respectively. The band alignment diagrams of a-IGZO films corresponding to the quantitative analyses are also given in Fig. 3(c). The results show that the *E*_g_ of a-IGZO film did not change noticeably, whereas the *E*_F_ increased from 2.73 eV to 3.01 eV after X-ray irradiation. Therefore, we can conclude that the increase of conductivity (or current level) during the X-ray irradiation is closely related to the increase in electron concentration in a-IGZO layer, which results in a negative *V*_TH_ shift of a-IGZO TFT.

**Fig. 4:**
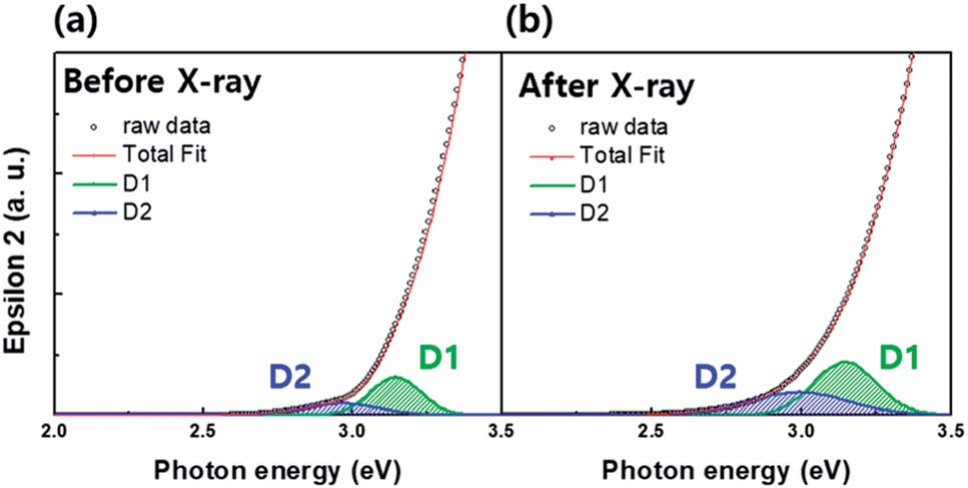
Spectroscopic ellipsometry (SE) spectra of the dielectric function (*ε*_2_) deconvoluted for two sub-gap states (denoted as D1 and D2) of the amorphous In–Ga–Zn–O (a-IGZO) film (a) before and (b) after X-ray irradiation. The two sub-gap states located near the conduction band minima were deconvoluted with a Gaussian fit. Both relative areas of the sub-gap states increased after X-ray irradiation.

Investigation of sub-gap states is a powerful way to understand the origin of electron concentration change in oxide semiconductors.^6–8^ The information regarding the a-IGZO sub-gap states near conduction band minimum (CBM) can be also obtained from the SE analysis. Fig. 4(a) and (b) compare the SE spectra changes in absorption coefficient in a-IGZO film before and after the X-ray irradiation. The band edge states were deconvoluted with Gaussian fit into two sub-gap states (denoted as D1 and D2). The spectra reveal the increase in relative areas of both D1 and D2 states after X-ray irradiation.

To date, extensive studies with respect to oxide semiconductors have been conducted, and there is agreement that hydrogen and oxygen vacancies are the main sources that affect the conductivity. Particularly, hydrogen has a strong influence on the D1 state, whereas the D2 state is dependent on the process parameters of the oxide semiconductors.^16,17^ Since we performed the SE analysis on the same sample, the only difference is the existence/nonexistence of X-ray irradiation. For this reason, it is obvious that the increase in relative area of the D1 and D2 states is caused by X-ray irradiation and strongly associated with the electron generation in a-IGZO film, which will be discussed later in more detail.

As mentioned previously, hydrogen can significantly affect the electrical characteristics of oxide-based TFTs. Therefore, a number of in-depth studies have been conducted on the role of hydrogen in the oxide semiconductors under various conditions. In general, hydrogen has two types of donor-like states near the CBM in the forms of H_i_^+^ in which H is bonded to an oxygen atom, and H_O_^+^ in which H is located at the oxygen vacancy.^12–15^ In addition to the donation of electrons, it is known that hydrogen can also enhance the electron mobility by way of passivating the electron trapping sites generated during the fabrication process.^12,18^

However, the effect of hydrogen in the X-ray environment has not been studied to date. Therefore, we investigated the behavior of hydrogen under X-ray irradiation by following possible hydrogen incorporation paths: (i) released hydrogen from adjacent hydrogen-rich layers near the a-IGZO film, *e.g.*, buffer layer and/or gate insulator during X-ray irradiation,^19,20^ and (ii) radical-form hydrogen generated by dissociation of H_2_O molecules during X-ray irradiation either in the air or on the device surface that can incorporate into the a-IGZO film.^21^ To investigate the hydrogen incorporation paths, we prepared two types of devices. In device A, a-IGZO film was deposited on the plasma-enhanced chemical vapor deposition (PECVD) grown SiN_*X*_/SiO_2_ buffer layer. PECVD grown SiN_*X*_ and SiO_2_ use silane (SiH_4_) and ammonia (NH_3_), SiH_4_ and nitrous oxide (N_2_O) as reacting gases, respectively.^22^ For this reason, a large amount of hydrogen can be resolved in the buffer layer. For device B, a-IGZO film was deposited directly on the bare substrate. Both devices were then exposed to an X-ray dose of 100 Gy in air and in an ambient vacuum to investigate the hydrogen effect not only inside the device but also under ambient conditions.

**Fig. 5:**
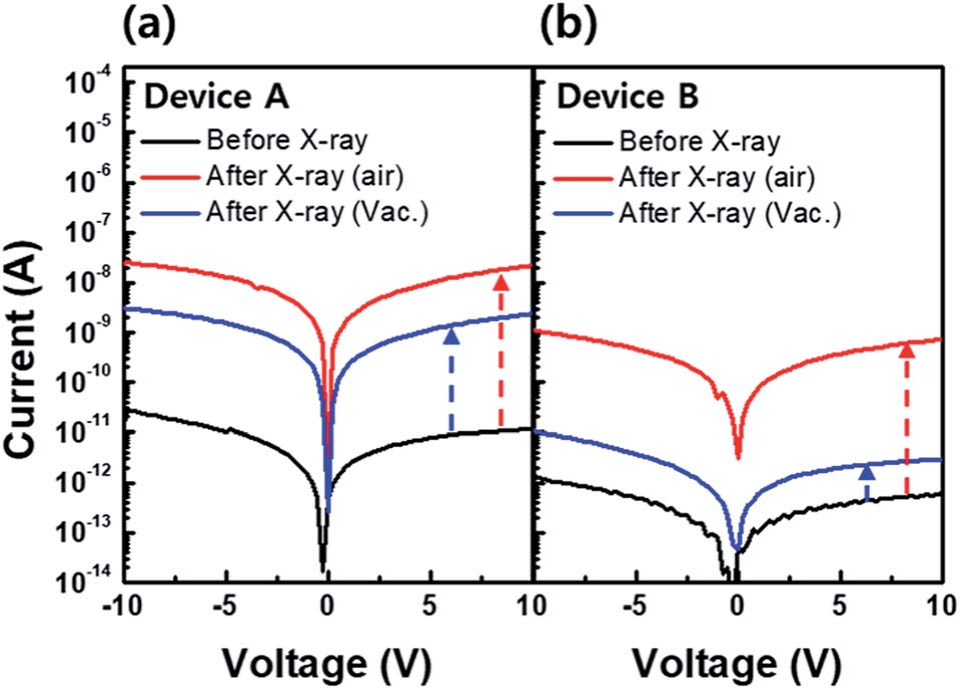
Current level variation of (a) device A (with hydrogen rich buffer layer) and (b) device B (without buffer layer) before (black line) and after X-ray irradiation in air (red line) and ambient vacuum (blue line). Device A shows more increase in current level after X-ray irradiation in air and ambient vacuum compared to that in device B. Both devices show less increase in current level in ambient vacuum irradiation compared to air ambient irradiation.

Fig. 5(a) and (b) exhibit the current level changes after X-ray irradiation in air and in a vacuum for device A (with buffer layer) and device B (without buffer layer), respectively. The initial current level (black line) of device A is about one order of magnitude higher than that of device B. This difference is due to the higher amount of hydrogen diffused from the buffer layer to the a-IGZO layer in device A during the post annealing process at 350 °C. Results show that the current level in both devices A and B after X-ray irradiation in ambient air (red line) increased by about three orders of magnitude. Such a jump in current level is an effect of X-ray irradiation. It also explains the area increase in hydrogen related to the D1 state in SE analysis after X-ray irradiation in Fig. 4. Interestingly, the increase in current level is less in the case of X-ray irradiation in the ambient vacuum (blue line) than that in ambient air in the both devices. Because the hydrogen sources from the air or the device surface can be excluded during the X-ray irradiation in ambient vacuum, there is a lower current level increase. In addition, the current level increase by X-ray irradiation in air and in ambient vacuum in device B is far less than that in device A. Since the only difference between the two devices is existence of buffer layer containing hydrogen, it suggests that hydrogen in the buffer layer plays an important role in electron generation during X-ray irradiation.

Given that the electrical characteristic that we evaluated is the current level or conductivity, it is thought that the enhanced conductivity is a combined effect of electron generation and defect passivation of hydrogen. Hence, the mobility increase due to the passivation effect would be partially added to the conductivity increase in the a-IGZO film during X-ray irradiation.

In addition to the hydrogen effect on the conductivity, another important factor to be considered is oxygen vacancy related defects, which depend on the process parameter. It is well known that one oxygen vacancy can release two electrons in oxide semiconductors through an oxygen vacancy ionization reaction such as V_O_ → V_O_^2+^ + 2e^−^. The ionization process can be accelerated under light illumination, which is so-called the persistent photocurrent (PPC) effect.^9–11^ However, the reverse reaction of the PPC effect is seldom observed due to the relatively large activation energy in the reverse reaction; and a number of experimental and calculation results show that the conductivity change is not fully recovered.^23–25^

Therefore, the increased relative area of the D2 state in the SE analysis is attributed to the V_O_^2+^ generation assisted by X-ray irradiation, which is another source that leads to increases in electron concentration.

**Fig. 6:**
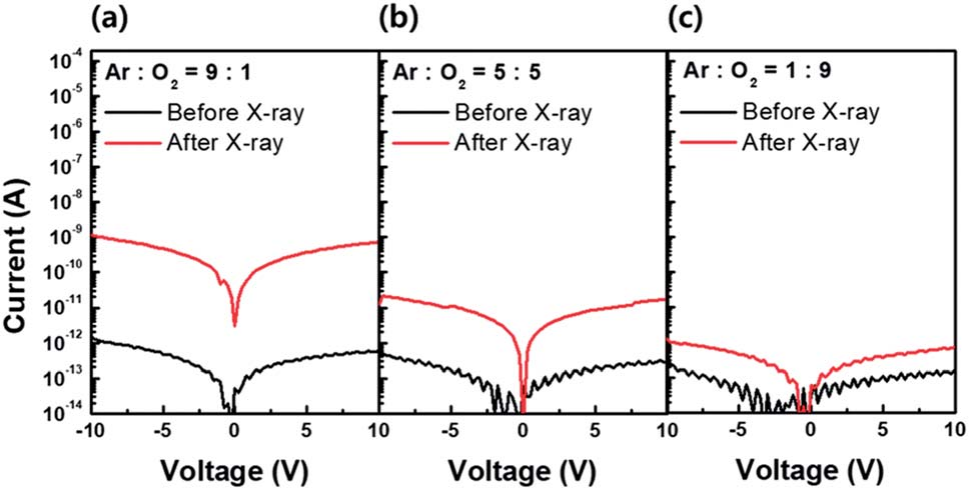
Current levels of amorphous In–Ga–Zn–O (a-IGZO) film before (black line) and after (red line) X-ray irradiation in three different a-IGZO active layers deposited with different Ar : O_2_ gas ratio: (a) 9 : 1, (b) 5 : 5, and (c) 1 : 9. The effect of X-ray irradiation on the increase in current level becomes less significant with increases in oxygen partial pressure. We confirmed that oxygen vacancy dependent PPC effect attributes to the electron generation which results in negative *V*_TH_ shift.

As explained previously, the PPC effect is dependent on the neutral oxygen vacancy concentration (V_O_). Therefore, to confirm the PPC effect, we prepared three a-IGZO films with different oxygen vacancy concentrations by changing the Ar : O_2_ gas ratio during the a-IGZO film deposition. Fig. 6(a), (b), and (c) show current levels before and after X-ray irradiation in three a-IGZO films deposited with Ar : O_2_ gas ratios of 9 : 1, 5 : 5, and 1 : 9, respectively. The three different samples before X-ray irradiation (black line) show a slight decrease in current level as the oxygen partial pressure increases. Considering the other process parameters as fixed, except oxygen partial pressure, a slight current level variation in the samples before X-ray irradiation is due to the different initial oxygen vacancy concentration. However, after X-ray irradiation (red line), the difference in current level becomes obvious and it decreases as the oxygen partial pressure increases. The X-ray irradiation presumably accelerates the ionization of the V_O_, and the difference in current level becomes more obvious in the sample with higher V_O_.

**Fig. 7:**
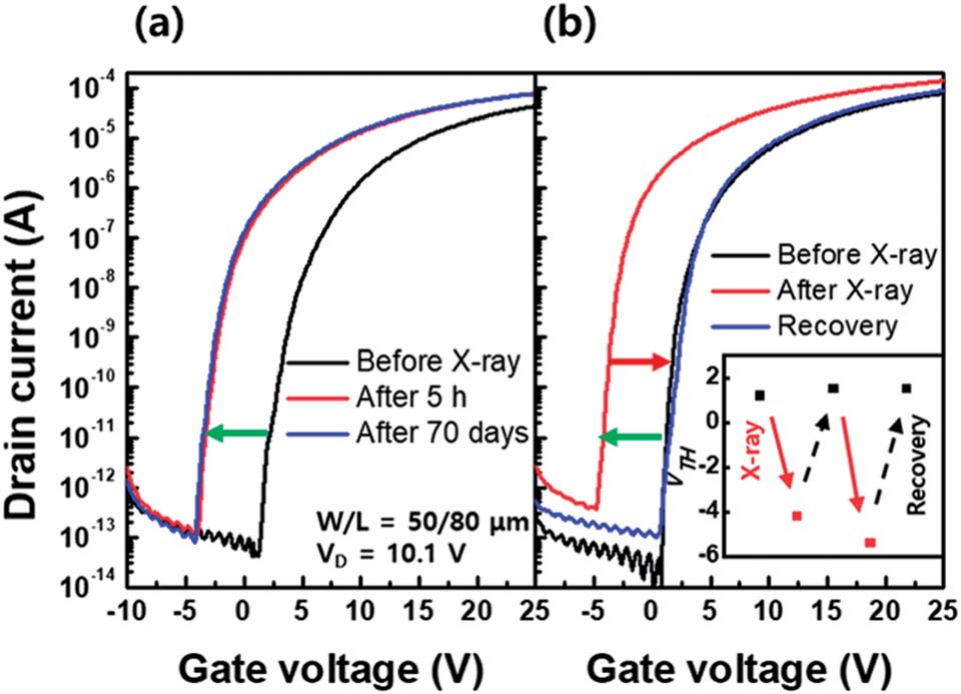
Transfer characteristics of an amorphous In–Ga–Zn–O (a-IGZO) thin film transistor (TFT) (a) before, 5 h after, and 70 days after X-ray irradiation. (b) Recovery behavior of transfer characteristics of the a-IGZO TFT by a thermal annealing process for 1 h at 350 °C. The *V*_TH_ shift through repetitive X-ray irradiation and annealing process (inset).

Fig. 7(a) shows the transfer characteristics of the a-IGZO TFT before, 5 h after, and 70 days after X-ray irradiation. As shown in Fig. 7(a), the negative *V*_TH_ shift in the a-IGZO TFT after X-ray irradiation is not recovered even after 70 days. It is reported that the migration barrier (∼2.5 eV) of hydrogen as a form of H_O_^+^ high and is difficult to be desorbed from the a-IGZO film at room temperature.^26^ On the other hand, another possible form of hydrogen, H_i_^+^, is movable even at room temperature and can be removed from the a-IGZO film as H_2_ molecules.^27^ Therefore, the *V*_TH_ shift even after 70 days is likely associated with the H_O_^+^ state. In addition, another interpretation for the Fig. 7(a) is the activation energy required in transition from the V_O_^2+^ state to V_O_ state in the PPC effect is relatively high.^9–11,23–25^

One way to recover the negative *V*_TH_ shift of the a-IGZO TFT is, therefore, to supply enough thermal energy. Fig. 7(b) shows fully recovered *V*_TH_ in the a-IGZO TFT to its initial state after the annealing process for 1 h at 350 °C. The inset in Fig. 7(b) demonstrates that the recovery by thermal annealing is repetitive.

## Conclusions

In this study, we evaluated the effects of X-ray irradiation on the electrical characteristics of the bottom-gated a-IGZO TFT and identified major causes that lead to negative *V*_TH_ shift in the transfer characteristics. The Fermi energy change suggests that such a degradation of electrical characteristics of TFT is caused by an increase in electron concentration in the active layer during X-ray irradiation. In relation with the electron generation, sub-gap states of D1 and D2 states near the conduction band were analyzed by SE analysis. The increase in relative area of the D1 state is associated with hydrogen incorporation, and we confirmed the donor-like hydrogen behavior under X-ray irradiation. Furthermore, it is believed that the H_O_^+^ state is responsible for the irreversibility in *V*_TH_ shift under X-ray irradiation. In addition, from the experimental results of the a-IGZO films with controlled oxygen vacancy concentration, we also found that the relative area increase of the D2 state is related to the electron generation by the PPC effect. As a result, the increased conductivity of the a-IGZO active layer after X-ray irradiation which leads to negative *V*_TH_ shift is due to the combined effect of the hydrogen incorporation and the PPC. Therefore, to minimize the *V*_TH_ shift of the oxide-based TFTs under X-ray irradiation techniques may be applied to minimize the hydrogen incorporation to the active layer or to control the oxygen vacancy concentration.

## Conflicts of interest

There are no conflicts to declare.

## Supplementary Material

RA-009-C9RA03053K-s001
