# Supplementary material for: Negative threshold voltage shift in an a-IGZO thin film transistor under X-ray irradiation
Source: RSC Adv. 2019 Jul 3;9(36):20865–70. doi: 10.1039/c9ra03053k (PMC9065737; doi:10.1039/c9ra03053k)
Supplement: RA-009-C9RA03053K-s001 [file RA-009-C9RA03053K-s001.pdf]

Electronic Supplementary Material (ESI) for RSC Advances.
This journal is © The Royal Society of Chemistry 2019

Supplementary information

Negative threshold voltage shift in an a-IGZO thin film transistor under X-ray irradiation

Dong-Gyu Kim[a], Jong-Un Kim[a], Jun-Sun Lee[a], Kwon-Shik Park[b], Youn-Gyoung Chang[b], Myeong-Ho Kim[a]* and Duck-Kyun Choi[a]*

[a] Division of Materials Science and Engineering, Hanyang University, 222, Wangsimni-ro, Seongdong-gu, Seoul, Republic of Korea

[b]Research and Development Center, LG Display Co., Ltd., 30, Magokjungang 10-ro, Gangseo-gu, Seoul, Republic of Korea.

Figures S1 (a) and (b) show the XPS spectra of the O1s chemical state before and after X-ray irradiation of an a-IGZO film, respectively. These spectra were deconvoluted through a Gaussian fit. The binding energy peaks reveal fully oxidized oxygen (M-O) at 529.7 ± 0.1 eV, an oxygen vacancy (V_O) at 530.7 ± 0.1 eV, and oxygen in the hydroxide state (M-OH) at 531.7 ± 0.1 eV. The peaks related to oxygen vacancy did not change significantly before and after X-ray irradiation, the percentage oxygen vacancies being 25.8% and 26.1% before and after X-ray irradiation, respectively. These results suggest that the cause of increased electron concentration during X-ray irradiation of a-IGZO film is not strongly related to the formation of oxygen vacancies. We estimated these results are attributed to the high permeability of X-ray irradiation with high photon energy.

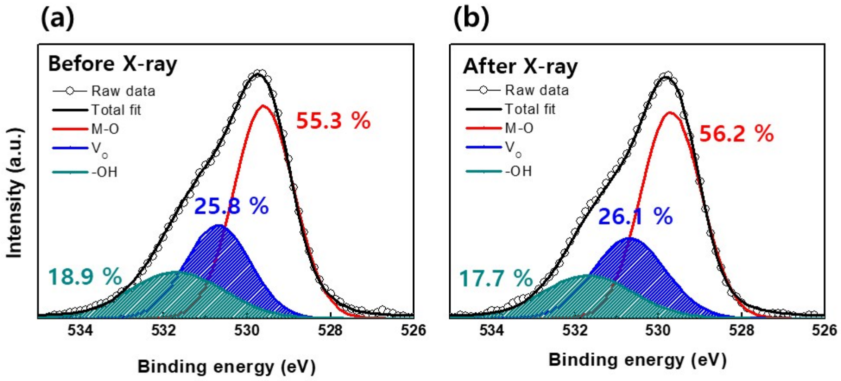

Figure S1. X-ray photoelectron spectroscopy (XPS) spectra of the O1s chemical state for the In-Ga-Zn-O (a-IGZO) film (a) before and (b) after X-ray irradiation. The binding energy states are related to fully oxidized oxygen (529.7 ± 0.1 eV), oxygen vacancy (530.7 ± 0.1 eV), and hydroxide (531.7 ± 0.1 eV), with the three binding states deconvoluted by Gaussian fits.
